# Methicillin-Resistant *Staphylococcus aureus* Carriage among Neonate Mothers, Healthcare Workers, and Environmental Samples in Neonatal Intensive Care Units: A Systematic Review

**DOI:** 10.1155/2024/5675786

**Published:** 2024-04-08

**Authors:** Nene Kaah Keneh, Sebastien Kenmoe, Arnol Bowo-Ngandji, Jane-Francis Tatah Kihla Akoachere, Hortense Gonsu Kamga, Roland Ndip Ndip, Jean Thierry Ebogo-Belobo, Cyprien Kengne-Ndé, Donatien Serge Mbaga, Nicholas Tendongfor, Jean Paul Assam Assam, Lucy Mande Ndip, Seraphine Nkie Esemu

**Affiliations:** ^1^Department of Microbiology and Parasitology, University of Buea, Buea, Cameroon; ^2^Laboratory for Emerging Infectious Diseases, University of Buea, Buea, Southwest Region, Cameroon; ^3^Department of Microbiology, The University of Yaounde I, Yaounde, Cameroon; ^4^Faculty of Medicine and Biomedical Sciences, The University of Yaounde I, Yaoundé, Cameroon; ^5^Center for Research in Health and Priority Pathologies, Institute of Medical Research and Medicinal Plants Studies, Yaounde, Cameroon; ^6^Epidemiological Surveillance, Evaluation and Research Unit, National AIDS Control Committee, Douala, Cameroon; ^7^Department of Public Health and Hygiene, University of Buea, P.O. Box 63, Buea, Cameroon

## Abstract

**Background:**

Methicillin-resistant *Staphylococcus aureus* (MRSA) is a significant cause of morbidity and mortality among neonates admitted to neonatal intensive care units (NICUs). The MRSA colonization of neonates, attributed to various sources, including mothers, healthcare workers, and environmental surfaces, can lead to severe infection, prolonged hospital stays, and even death, imposing substantial economic burdens. Given the pressing need to mitigate MRSA spread in these vulnerable environments, further examination of the subject is warranted. This systematic review is aimed at synthesizing available evidence on MRSA carriage proportions among mothers of newborns, healthcare workers, and environmental surfaces in NICUs. *Methodology*. We included observational studies published in English or French from database inception to March 21, 2023. These studies focused on MRSA in nonoutbreak NICU settings, encompassing healthy neonate mothers and healthcare workers, and environmental surfaces. Literature search involved systematic scanning of databases, including Medline, Embase, Web of Science, Global Health, and Global Index Medicus. The quality of the selected studies was assessed using the Hoy et al. critical appraisal scale. The extracted data were summarized to calculate the pooled proportion of MRSA positives, with a 95% confidence interval (CI) based on the DerSimonian and Laird random-effects model.

**Results:**

A total of 1891 articles were retrieved from which 16 studies were selected for inclusion. Most of the studies were from high-income countries. The pooled proportion of MRSA carriage among 821 neonate mothers across four countries was found to be 2.1% (95% CI: 0.3-5.1; *I*^2^ = 76.6%, 95% CI: 36.1-91.5). The proportion of MRSA carriage among 909 HCWs in eight countries was determined to be 9.5% (95% CI: 3.1-18.4; *I*^2^ = 91.7%, 95% CI: 87.1-94.6). The proportion of MRSA carriage among HCWs was highest in the Western Pacific Region, at 50.00% (95% CI: 23.71-76.29). In environmental specimens from five countries, a pooled proportion of 16.6% (95% CI: 3.5-36.0; *I*^2^ = 97.7%, 95% CI: 96.6-98.4) was found to be MRSA-positive.

**Conclusion:**

With a significant heterogeneity, our systematic review found high MRSA carriage rates in neonate mothers, healthcare workers, and across various environmental surfaces in NICUs, posing a potential risk of nosocomial infections. Urgent interventions, including regular screening and decolonization of MRSA carriers, reinforcing infection control measures, and enhancing cleaning and disinfection procedures within NICUs, are crucial. This trial is registered with CRD42023407114.

## 1. Introduction

Neonatal intensive care units (NICUs) offer specialized care to neonates suffering from severe health problems. In NICUs, methicillin-resistant *Staphylococcus aureus* (MRSA) can colonize neonates [1, 2]. As a result of MRSA colonization, neonates are at risk of infection and death, requiring prolonged hospital stays and incurring substantial economic costs [3–5]. MRSA was initially associated with hospital environments, with colonization rates reaching 50% in neonates in NICUs [6, 7]. Later, MRSA emerged in the community, causing concern at equally significant levels since outborn newborns admitted to the NICU were found to be already colonized [8, 9].

In the NICU environment, MRSA contamination is a major concern due to neonates' high susceptibility to colonization and infection. Many elements within the NICU have been identified as potential sources of MRSA transmission, including environmental surfaces, healthcare workers (HCWs), and mothers of newborns and the expressed breast milk (EBM) that they provide [10–14]. In the NICU, MRSA can be present on equipment, indoor settings, and even toys, contributing to newborn colonization. MRSA transmission in the NICU is primarily attributed to healthcare workers and neonate mothers. Inappropriate handling of EBM can lead to bacterial contamination, posing a health risk to neonates. A carriage of MRSA among healthcare professionals may result in the transmission of colonization or infection to neonates during treatment and routine contact with them [10, 15]. MRSA can persist for months on dry inanimate surfaces, especially under humid conditions and at low temperatures, which increases the risk of transmission within the NICU [16]. Many institutions closely monitor MRSA colonization in neonates, and universal decolonization protocols are often used to eliminate this problem [17–19].

To establish effective preventive measures, it is important to recognize MRSA presence among newborns, their mothers, healthcare workers, and environmental surfaces. The transmission of MRSA within NICUs is complex and not yet fully understood, with multiple sources likely contributing to MRSA spread, including healthcare workers, other patients, and family members. As NICUs are vulnerable environments and there is a pressing need to control MRSA spread, it is imperative to gain a comprehensive understanding of MRSA carriage among these various potential sources. In this systematic review, we aim to synthesize available evidence to provide a thorough overview of the issue. Our primary focus will be on the proportion of MRSA colonization in mothers of newborns, healthcare workers, and environmental samples within NICUs.

## 2. Methods

### 2.1. Review Protocol and Research Question

We conducted this systematic review in accordance with the Preferred Reporting Items for Systematic Reviews and Meta-Analyses (PRISMA) 2020 guidelines [20]. After formulating a research question, we prospectively submitted the review protocol to PROSPERO on March 21, 2023. This protocol includes detailed methodology, review questions, and eligibility criteria (registration number: CRD42023407114).

### 2.2. Inclusion and Exclusion Criteria

We considered observational or interventional studies published in English or French between the database creation and March 21, 2023. These studies included apparently healthy neonate mothers, HCWs, and environmental surfaces with a laboratory diagnosis of MRSA in nonoutbreak NICU settings. We excluded case reports, outbreak investigations, systematic reviews, meta-analyses, editorials, commentaries, articles with participants with clinical infection, and articles with participants other than neonate mothers, HCWs, or environmental surfaces, or those that lacked sufficient data or were inaccessible.

### 2.3. Literature Search

An author (SK) systematically searched databases (Medline, Embase, Web of Science, Global Health, and Global Index Medicus) to identify relevant articles that reported epidemiological data regarding MRSA from the beginning of the database to March 2023 (Table S1). The comprehensive search strategy employed MeSH Terms and keywords (MRSA AND NICU) in conjunction with Boolean operators “AND” and “OR.” The reference section of each shortlisted study was also examined to identify additional studies not identified during the initial database search.

### 2.4. Selection Process and Data Extraction

Two authors (ABN and SK) independently assessed the titles and abstracts remaining after eliminating duplicates to identify potentially eligible articles that should be reviewed in more detail based on the criteria for eligibility [21]. Both authors conducted full-text reviews independently, and disagreements were resolved through discussion and consensus. After evaluating the studies for inclusion, we extracted data onto a standardized Google Forms, including the following headings: first author names, countries, study period, participants (neonate mothers, healthcare staff, or environmental surfaces), MRSA detection assays, specimen types tested, MRSA molecular confirmations, sample size, and total number of MRSA positives.

### 2.5. Quality Assessment

To assess the quality of selected studies, two authors (ABN and SK) individually used the Hoy et al. critical appraisal scale for prevalence studies [22]. The quality assessment checklist can be found in Table S2.

### 2.6. Statistical Analysis

SK analyzed the extracted data from each of the selected studies using R (version 4.0.3, released on October 10, 2020, using the meta and metafor packages) [23, 24]. We calculated the pooled proportion of MRSA positives among neonate mothers, HCWs, and environmental surfaces, with a 95% confidence interval (CI) based on the DerSimonian and Laird random-effects model after a Freeman-Tukey double-arcsine transformation to stabilize variances [25]. Based on the WHO regions, we assessed the heterogeneity of the included studies using Cochran's *Q* test (chi-square test) or *I*^2^ statistics [26, 27]. We tested for publication bias using Egger's regression tests, with a *P* value of 0.10 indicating a statistically significant effect [28].

## 3. Results

### 3.1. Study Selection

**Figure 1:**
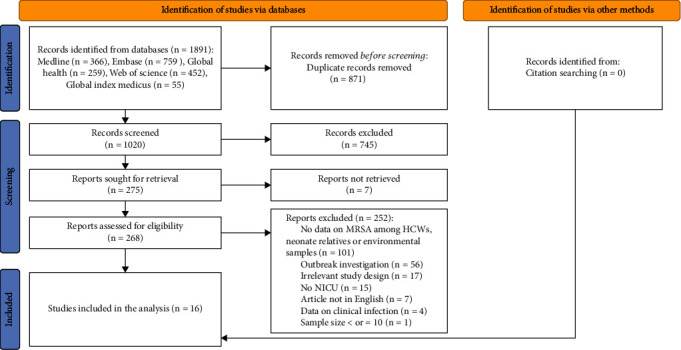
PRISMA diagram showing selection of studies.

We initially identified 1891 articles, distributed as follows: 366 from Medline, 759 from Embase, 259 from Global Health, 452 from Web of Science, and 55 from Global Index Medicus. After deduplication, 1020 unique articles remained further screening. Of these, 745 articles were deemed irrelevant and excluded, while 268 articles were selected for full-text assessment based on our established inclusion and exclusion criteria. Out of these, we excluded an additional 252 articles, primarily due to the lack of MRSA data related to healthcare workers, neonate mothers, or environmental surfaces, or because they focused solely on outbreak investigations. In the end, 16 articles were selected for inclusion in the qualitative synthesis and meta-analysis [29–44].  Figure 1 shows our article retrieval and screening process.

### 3.2. Study Characteristics

**Figure 2:**
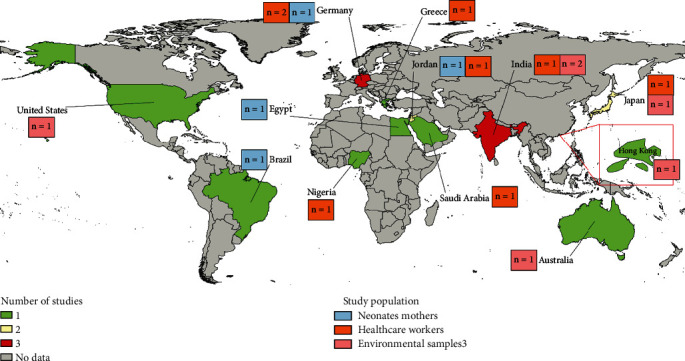
Location of included studies reporting MRSA carriage among neonate mothers, healthcare workers, and environmental samples in neonatal intensive care units.

Most of the studies were based in high-income countries (9 out of 16) and located in Europe (4 out of 16) and the Western Pacific Region (3 out of 16) (Figure 2 and Table S3). HCWs were the most studied group (8 out of 16) [29, 31, 34, 35, 39–41, 44], followed by environmental samples (6 out of 16) [30, 32, 35, 37, 38, 43] and neonate mothers (4 out of 16) [31, 33, 36, 42]. The reasons for testing for MRSA colonization in the included studies vary significantly, ranging from routine surveillance to specific medical interventions and responses to identified issues, such as pre- and posthand hygiene interventions, cleaning measures, admission due to preterm birth, and responses to ongoing MRSA detections in neonates. The detection methods employed across the included studies encompass both culture techniques and PCR analysis, with one study utilizing the API20E system for bacterial identification. Antibiotic susceptibility was predominantly assessed using the disk diffusion method with cefoxitin, adhering to the guidelines from the Clinical and Laboratory Standards Institute (CLSI) in two studies, while another study followed the European Committee on Antimicrobial Susceptibility Testing (EUCAST) guidelines. Sample types frequently collected were nasal/nares (8 out of 16) and surfaces (6 out of 16). MRSA confirmation was only performed in a fraction of the studies (3 out of 16) using mecA, nuc, or both genes. Some studies had a low risk of bias (3 out of 16), but most presented a moderate risk of bias (13 out of 16) (Table S4).

The base map was taken from https://www.naturalearthdata.com and modified with QGIS software version 3.16.0-Hannover.

### 3.3. Proportion of MRSA Carriage in Neonate Mothers in Neonatal Intensive Care Units

**Figure 3:**
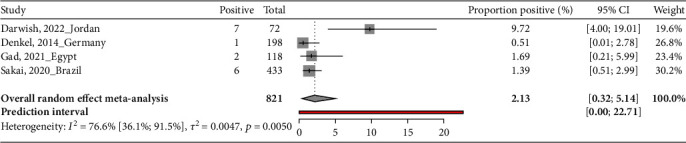
Proportion of MRSA carriage in neonate mothers in neonatal intensive care units.

This systematic review evaluates the proportion of MRSA carriage in 821 neonate mothers across four countries. In Jordan, a study involved 72 participants, revealing that 7 were colonized with MRSA, a proportion of 9.72% (95% CI: 4.00-19.01) [31]. Between May 2012 and June 2013, a German study assessed 198 participants and found only one mother colonized with MRSA, a prevalence of 0.51% (95% CI: 0.01-2.78) [33]. In Egypt, a study conducted from January to December 2019 tested 118 expressed breast milks, revealing that two were MRSA carriers, amounting to a proportion of 1.69% (95% CI: 0.21-5.99) [36]. In Brazil, a study conducted from January 2014 to February 2018 examined 433 participants and found six mothers colonized with MRSA, a prevalence of 1.39% (95% CI: 0.51-2.99) [42]. The pooled proportion of MRSA carriage among neonate mothers was 2.1% (95% CI: 0.3-5.1) (Table 1 and Figure 3).

### 3.4. Proportion of MRSA Carriage in Healthcare Workers in Neonatal Intensive Care Units

**Figure 4:**
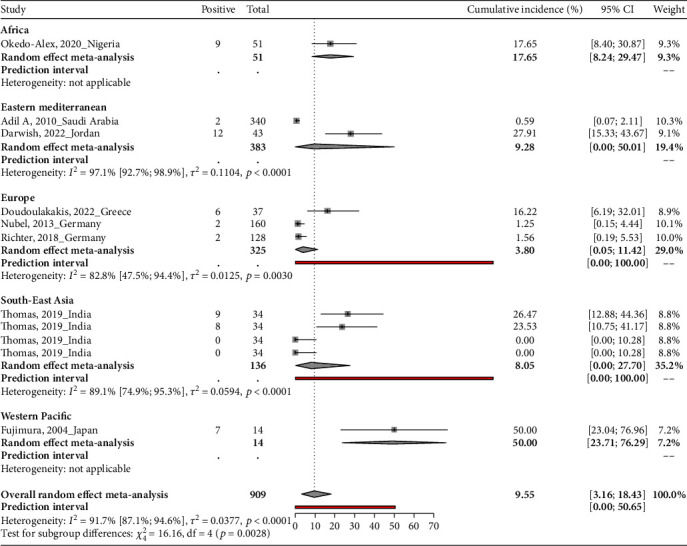
Proportion of MRSA carriage in healthcare workers in neonatal intensive care units.

This systematic review assesses the proportion of MRSA carriage in 909 healthcare workers in eight countries. In Nigeria, a study found 9 out of 51 HCWs colonized with MRSA (17.65%, 95% CI: 8.40-30.87) [40]. From September 2005 to May 2006, a study conducted in Saudi Arabia among nurses and physicians detected 2 MRSA carriers out of 340 participants (0.59%, 95% CI: 0.07-2.11) [29]. In Jordan, the proportion of MRSA carriage among doctors and nurses was 27.91% (12 out of 43 participants, 95% CI: 15.33-43.67) [31]. A study conducted in Greece from January 2014 to December 2018 among general doctors and nurses reported a prevalence of 16.22% (6 out of 37 participants, 95% CI: 6.19-32.01) [34]. Two German studies conducted in February-August 2010 and 2016 found a proportion of 1.25% (95% CI: 0.15-4.44) and 1.56% (95% CI: 0.19-5.53), respectively [39, 41]. In India, a study conducted between July and August 2013 among general doctors and nurses tested MRSA prevalence pre- and posthand hygiene intervention [44]. Before handwashing, 9 out of 34 participants (26.47%, 95% CI: 12.88-44.36) were found to be colonized with MRSA. This proportion slightly decreased after handwashing to 23.53% (8 out of 34 participants, 95% CI: 10.75-41.17). Postintervention, before and after handwashing, no participants were found to be colonized (0 out of 34 participants, 95% CI: 0.00-10.28). A study in Japan from January to June 2001 reported the highest prevalence of 50.00% (7 out of 14 participants, 95% CI: 23.04-76.96) [35]. The pooled proportion of MRSA carriage among healthcare workers was 9.5% (95% CI: 3.1-18.4) (Table 1 and Figure 4). The differences in the proportion among HCWs in the WHO regions were statistically significant with a *P* value of < 0.001. The proportion of MRSA carriage among HCWs was highest in the Western Pacific Region, with a proportion of 50.00% (95% CI: 23.71-76.29), followed by Africa 17.65% (95% CI: 8.24-29.47), Eastern Mediterranean 9.28% (95% CI: 0.00-50.01), Southeast Asia 8.05% (95% CI: 0.00-27.70), and Europe 3.80% (95% CI: 0.05-11.42).

### 3.5. Proportion of MRSA Carriage in Environmental Surfaces in Neonatal Intensive Care Units

**Figure 5:**
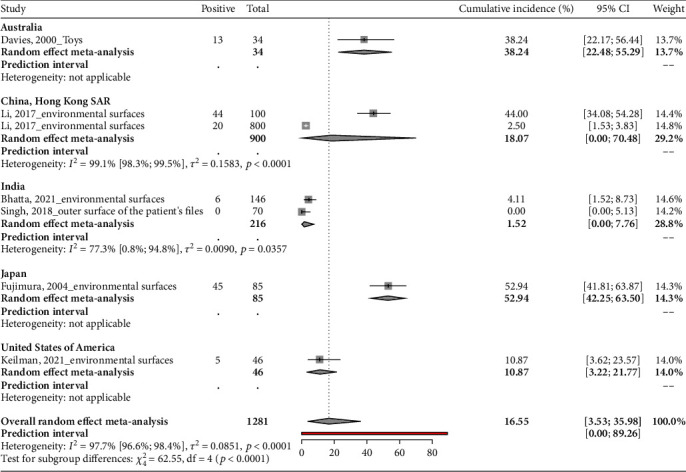
Proportion of MRSA carriage in environmental surfaces in neonatal intensive care units.

A total of 1281 environmental specimens from five countries were assessed for the presence of MRSA. In Japan, a high proportion of 52.94% (95% CI: 41.81-63.87) of diverse samples including incubators, sheets, and stethoscopes were MRSA-positive during the study period from January 2001 to June 2001 [35]. Hong Kong also had a high proportion of MRSA at 44.00% (95% CI: 34.08-54.28) on various environmental surfaces during the baseline period (no environmental cleaning measures) from January 2009 to December 2013 [38]. This proportion dropped to 2.50% (95% CI: 1.53-3.83) postintervention period after environmental cleaning measures. In Australia, 38.24% (95% CI: 22.17-56.44) of the sampled toys were contaminated [32]. The study in the USA identified a contamination proportion of 10.87% (95% CI: 3.62-23.57) on baby isolette stations, communal equipment, and physical plants [37]. In India, a relatively low contamination proportion of 4.11% (95% CI: 1.52-8.73) was found on various hospital equipment, with no MRSA found on the outer surface of the patient files [30]. There was a pooled proportion of 16.6% (95% CI: 3.5-36.0) of MRSA in environmental specimens (Table 1 and Figure 5).

### 3.6. Publication Bias and Heterogeneity

For neonate mothers, HCWs, and environmental samples, we found substantial heterogeneity with an *I*^2^ of 76.6% (95% CI: 36.1-91.5), 91.7% (95% CI: 87.1-94.6), and 97.7% (95% CI: 96.6-98.4), respectively (Table 1). The symmetry observed in the funnel plots for datasets concerning neonate mothers and environmental samples implied a lack of publication bias within these groups (Figures S1 and S2). Asymmetry in the funnel plots for the HCWs category showed potential publication bias (Figure S3). This was further confirmed by the nonsignificant Egger's publication bias test for neonate mothers (*P* = 0.316) and environmental samples (*P* = 0.134), and a significant result for the HCWs group (*P* = 0.004).

## 4. Discussion

The study is the first comprehensive systematic review about the prevalence of MRSA among neonate mothers, healthcare workers, and environmental surfaces. This systematic review synthesized the findings from 16 articles. The studies predominantly took place in high-income countries. In total, the review analyzed data from 821 neonate mothers across four countries and found a proportion of 2.1% (95% CI: 0.3-5.1). The review examined 909 healthcare workers across eight countries and found a much higher proportion of 9.5% (95% CI: 3.1-18.4). Notably, there were statistically significant differences in the proportion of healthcare workers across the different WHO regions. The highest proportion was in the Western Pacific Region with 50.00% (95% CI: 23.71-76.29), followed by Africa with 17.65% (95% CI: 8.24-29.47), Eastern Mediterranean with 9.28% (95% CI: 0.00-50.01), Southeast Asia with 8.05% (95% CI: 0.00-27.70), and Europe with 3.80% (95% CI: 0.05-11.42). In the analysis of 1281 environmental samples from five countries, the review found a proportion of 16.6% (95% CI: 3.5-36.0).

In this systematic review, we observed that the prevalence of MRSA carriage was 2.1% in mothers of neonates and 9.5% in healthcare workers. Comparing our results with literature data, in Iran, for example, a much higher rate at 32.8% (95% CI: 26.0-40.4) of nasal MRSA carriage among healthcare workers was reported from a systematic review of 22 studies [15]. In South Asia, another systematic review including studies conducted in nonoutbreak settings from 2000 to 2021 reported a similar MRSA carriage rate in healthcare workers at 9.23% (95% CI; 6.50%, 12.35%) [10]. Our systematic review identified a MRSA proportion of 16.6% (95% CI: 3.5-36.0) on surfaces in NICU. A systematic review in various settings, including hospitals, intensive care units, general wards, nursing homes, and long-term care facilities, reported that the MRSA prevalence on healthcare worker attire varies significantly [11]. The MRSA rates ranged between 1.3 and 14% (for studies focusing on gowns), between 4 and 79% (white coats), between 0 and 19.1% (Scrubs), between 3.5 and 19.1% (uniforms, both short- and long-sleeved), and between 2.5 and 32% (ties). Our findings fall within the reported range for these various types of attire, but our study focused specifically on environmental samples in NICUs, which may have different contamination dynamics than general hospital settings or long-term care facilities due to the unique vulnerabilities of neonates. In our study, the reported prevalence varied significantly within each group of neonate mothers, HCWs or environmental samples, likely because of differences in sampling methods, culture techniques, and antibiotic management. Several factors have been identified as contributing to the contamination of expressed breast milk, including improper handwashing, lack of proper breast hygiene, and the methods used for storage and transport [14, 45]. The rate of MRSA carriage among healthcare workers is known to be significantly influenced by the control measures implemented, such as hand hygiene practices, the use of personal protective equipment (e.g., gowns, gloves, and masks), and the extent of awareness raising and training on infections control measures [46]. Research showed that nurses are more likely to be carriers of MRSA than other types of healthcare workers (e.g., doctors, laboratory staff, and students), largely due to their increased frequency of patient contact [10, 15]. Additionally, clothing worn by healthcare workers may become contaminated with MRSA [11]. Certain items, such as white coats and ties, have been more frequently identified as sources of contamination, suggesting also that short-sleeved uniforms may be more advantageous. The frequency and methods of washing these uniforms, which are often cleaned at home by healthcare workers, could potentially contribute to the level of MRSA contamination. Studies have demonstrated that objects and instruments most frequently touched in healthcare settings are more susceptible to MRSA contamination [12].

Our review does have notable limitations. Primarily, it only considers published studies, which may introduce publication bias into our findings. The significant heterogeneity observed within our systematic review underlines a limitation in the interpretability and generalizability of our findings. The relatively small number of studies addressing subcategories of health workers, various types of environmental samples, and diverse geographic regions restricts our ability to make dependable inferences. The variation in testing rationale can influence the apparent prevalence of MRSA carriage and should be carefully considered when interpreting the review's findings. Furthermore, the underrepresentation of studies from developing countries limits the global applicability of our findings. The skewed representation may potentially compromise the generalization of our results at a worldwide scale. There are several areas where additional research could augment and refine our current understanding of MRSA carriage in NICUs. These areas include a deeper investigation into MRSA carriage, particularly in resource-limited settings. A greater focus on the examination of expressed breast milk is also necessary, along with an in-depth study of healthcare worker subcategories, with a primary emphasis on nurses. Furthermore, frequent instruments or objects, such as catheters, warrant additional scrutiny. We need to understand better the interplay between contamination of healthcare workers, parents of newborns, and environmental surfaces and how these factors contribute to the colonization or infection of neonates. The use of genomic data could be instrumental in reconstructing MRSA transmission events, offering potentially crucial insights into the spread of this bacterium [47]. Additionally, research should be directed towards understanding the impact of surface cleaning in microbial decontamination. We should investigate the behavioral determinants influencing adherence to infection control measures, particularly hand hygiene.

In our review, we observed a high carriage rate of MRSA in mothers of neonates, healthcare workers, and across various environmental samples in NICUs. This situation presents a significant risk and potentially contributes to the spread of nosocomial infections. As such, urgent interventions are required to address this risk within NICUs. Key among these interventions includes regular screening and decolonization of neonate family members and healthcare workers carrying MRSA in NICUs [48]. The strengthening of infection control measures is also crucial, which should involve the reinforcement of contact precautions and hand hygiene practices [49]. Moreover, the enhancement of cleaning and disinfection procedures within the NICU environment is of utmost importance. Sensitizing both family members of newborns and healthcare workers about appropriate sanitation measures, and the specific risks associated with expressed breast milk, can further help mitigate this risk [14, 45, 46]. Additionally, there is a clear need for comprehensive guidelines concerning the use of expressed breast milk for vulnerable children in NICUs.

## Figures and Tables

**Table 1:** Results of meta-analysis of MRSA carriage among neonate mothers, healthcare workers, and environmental samples in neonatal intensive care units.

	Prevalence. (%) [95% CI]	95% prediction interval	*N* studies	*N* participants	*H* ^¶^ [95% CI]	*I* ^2^^§^ [95% CI]	*P* heterogeneity	*P* value Egger test
Neonate mothers	2.1 [0.3-5.1]	[0-22.7]	4	821	2.1 [1.3-3.4]	76.6 [36.1-91.5]	0.005	0.316
HCWs	9.6 [3.2-18.4]	[0-50.6]	11	909	3.5 [2.8-4.3]	91.7 [87.1-94.6]	<0.001	0.004
Environmental samples	16.6 [3.5-36]	[0-89.3]	7	1281	6.6 [5.5-8]	97.7 [96.6-98.4]	<0.001	0.134

CI: confidence interval; *N*: number; 95% CI: 95% confidence interval; NA: not applicable. ^¶^*H* is a measure of the extent of heterogeneity, a value of *H* = 1 indicates homogeneity of effects, and a value of *H* > 1 indicates a potential heterogeneity of effects. ^§^*I*^2^ describes the proportion of total variation in study estimates that is due to heterogeneity; a value >50% indicates the presence of heterogeneity.

## Data Availability

All relevant data are within the paper and its supporting files.

## Supplementary Materials

Supplementary Materials Table S1: Search strategy. Table S2: Items for risk of bias assessment. Table S3: Individual characteristics of included studies. Table S4: Risk of bias assessment. Figure S1: Funnel chart for publications of the proportion of MRSA carriage in neonate mothers in neonatal intensive care units. Figure S2: Funnel chart for publications of the proportion of MRSA carriage in healthcare workers in neonatal intensive care units. Figure S3: Funnel chart for publications of the proportion of MRSA carriage in environmental surfaces in neonatal intensive care units.
