# Supplementary material for: Methicillin-Resistant Staphylococcus aureus Carriage among Neonate Mothers, Healthcare Workers, and Environmental Samples in Neonatal Intensive Care Units: A Systematic Review
Source: Biomed Res Int. 2024 Apr 8;2024:5675786. doi: 10.1155/2024/5675786 (PMC11018372; doi:10.1155/2024/5675786)
Supplement: Supplementary Materials — Table S1: Search strategy. Table S2: Items for risk of bias assessment. Table S3: Individual characteristics of included studies. Table S4: Risk of bias assessment. Figure S1: Funnel chart for publications of the proportion of MRSA carriage in neonate mothers in neonatal intensive care units. Figure S2: Funnel chart for publications of the proportion of MRSA carriage in healthcare workers in neonatal intensive care units. Figure S3: Funnel chart for publications of the proportion of MRSA carriage in environmental surfaces in neonatal intensive care units. [file 5675786.f1.pdf]

Supplementary Materials

Table S1: Search strategy

Database		Search done on Jannuary 5th, 2023	Items
**Medline (Ovid)**	1	exp Methicillin-Resistant Staphylococcus aureus/	19041
	2	(Methicillin-Resistant Staphylococcus aureus or MRSA).mp.	34351
	3	exp Sepsis/ or exp Neonatal Sepsis/ or exp Bacteremia/ or exp Endotoxemia/ or exp Shock, Septic/ or exp Hemorrhagic Septicemia/	139775
	4	(Sepsis or Neonatal Sepsis or Bacteremia or Endotoxemia or Shock, Septic or Hemorrhagic Septicemia or Bloodstream Infection or Bloodstream Infections or Pyemia or Pyemias or Pyohemia or Pyohemias or Pyaemia or Pyaemias or Septicemia or Septicemias or Blood Poisoning or Blood Poisonings or Severe Sepsis).mp.	193563
	5	exp Intensive Care Units, Neonatal/	17833
	6	(Newborn Intensive Care Unit or Newborn Intensive Care Units or Neonatal Intensive Care Unit or Neonatal Intensive Care Units or NICU or Neonatal ICU or Neonatal ICUs or Newborn ICU or Newborn ICUs).mp.	24242
	7	1 or 2	34351
	8	3 or 4	211811
	9	5 or 6	30453
	10	7 and 8 and 9	102
**Embase (Ovid)**	1	exp methicillin resistant Staphylococcus aureus/	55373
	2	(methicillin resistant Staphylococcus aureus or MRSA).mp.	73152
	3	exp bacteremia/ or exp bloodstream infection/ or exp endotoxemia/ or exp hemorrhagic septicemia/ or exp newborn sepsis/ or exp pyemia/ or exp sepsis/ or exp septic shock/ or exp septicemia/ or exp staphylococcal bacteremia/	350333
	4	(bacteremia or blood poisoning or blood poisonings or bloodstream infection or bloodstream infections or endotoxemia or hemorrhagic septicemia or neonatal sepsis or newborn sepsis or pyaemia or pyaemias or pyemia or pyemias or pyohemia or pyohemias or sepsis or septic shock or septicemia or septicemias or severe sepsis or staphylococcal bacteremia).mp.	402811
	5	exp neonatal intensive care unit/ or exp newborn intensive care/	48134
	6	(neonatal intensive care unit or newborn intensive care or Newborn Intensive Care Units or Neonatal Intensive Care Unit or Neonatal Intensive Care Units or NICU or Neonatal ICU or Neonatal ICUs or Newborn ICU or Newborn ICUs).mp.	63228
	7	1 or 2	73153
	8	3 or 4	415821
	9	5 or 6	63228
	10	7 and 8 and 9	328
**Global Health (Ovid)**	1	(Methicillin-Resistant Staphylococcus aureus or methicillin resistant Staphylococcus aureus or MRSA).mp.	22121
	2	(bacteremia or blood poisoning or blood poisonings or bloodstream infection or bloodstream infections or endotoxemia or hemorrhagic septicemia or neonatal sepsis or newborn sepsis or pyaemia or pyaemias or pyemia or pyemias or pyohemia or pyohemias or sepsis or septic	62406

		shock or septicemia or septicemias or severe sepsis or staphylococcal bacteremia).mp.	
	3	(Neonatal ICU or Neonatal ICUs or neonatal intensive care unit or Neonatal Intensive Care Units or Newborn ICU or Newborn ICUs or Newborn Intensive Care Unit or Newborn Intensive Care Units or newborn intensive care or NICU).mp.	6472
	4	1 and 2 and 3	71
**Web of Science**	1	Topic = (((Methicillin-Resistant Staphylococcus aureus OR methicillin resistant Staphylococcus aureus OR MRSA) AND (bacteremia or blood poisoning or blood poisonings or bloodstream infection or bloodstream infections or endotoxemia or hemorrhagic septicemia or neonatal sepsis or newborn sepsis or pyaemia or pyaemias or pyemia or pyemias or pyohemia or pyohemias or sepsis or septic shock or septicemia or septicemias or severe sepsis or staphylococcal bacteremia)) AND (Neonatal ICU OR neonatal intensive care unit OR Newborn ICU OR Newborn Intensive Care Unit OR newborn intensive care OR NICU)) AND (Newborn OR Neonate)	109

Table S2: Items for risk of bias assessment

	Yes (1)	No (0)	Unclear (0)	Not applicable (0)
1. Was the study's target population a close representation of the national population in relation to relevant variables, e.g. age, sex, occupation?				
2. Was the sampling frame a true or close representation of the target population?				
3. Was some form of random selection used to select the sample, OR was a census undertaken?				
4. Was the likelihood of non-response bias minimal?				
5. Were data collected directly from the subjects (as opposed to a proxy)?				
6. Was an acceptable case definition used in the study?				
7. Was the study instrument that measured the parameter of interest shown to have reliability and validity (if necessary)?				
8. Was the same mode of data collection used for all subjects?				
9. Was the length of the shortest prevalence period for the parameter of interest appropriate?				
10. Were the numerator(s) and denominator(s) for the parameter of interest appropriate?				
Summary item on the overall risk of study bias				
Interpretation of the risk of bias tool				

- 7-10: Low risk of bias
- 4-6: Moderate risk of bias
- 0-3: High risk of bias

Table S3: Individual characteristics of included studies

Authors	Countries	Study period	Samples categories	Reason to test for colonization	Microbiological methods	Sample types	No. of participants	No. of participants with MRSA	Proportion positive [95 % CI]
Adil A et al., 2010	Saudi Arabia	Sep/2005-May/2006	Nurses and Physicians	Screening of staff members who are in close contact with patients	Culture; Disk diffusion method.	Nasal/ Nares	340	2	0.59 [0.07; 2.11]
Bhatta et al., 2021	India	Unclear/Not reported	Ambu bag, Bedside locker, BP Machine, Door handles, Hood box, Incubator, Laryngoscope, Mothers' bed, Phototherapy bed, Radiant warmer, Station counter, Sterilizer, Stethoscope, Suction tip, Telephone set, Ventilator, Wall BPL monitor, Weighing machine	Routine surveillance study	Culture; Disk diffusion method using cefoxitin	Surfaces sampled	146	6	4.11 [1.52; 8.73]
Darwish et al., 2022	Jordan	Unclear/Not reported	doctors and nurses	Routine surveillance study	Culture; Disk diffusion method using cefoxitin, PCR	Nasal/ Nares	43	12	27.91 [15.33; 43.67]
Darwish et al., 2022	Jordan	Unclear/Not reported	Neonate mothers	Routine surveillance study	Culture; Disk diffusion method using cefoxitin, PCR	Nasal/ Nares	72	7	9.72 [4.00; 19.01]

Davies et al., 2000	Australia	Unclear/Not reported	Toys	Routine surveillance study	Culture	Surfaces sampled	34	13	38.24 [22.17; 56.44]
Denkel et al., 2014	Germany	May/2012-Jun/2013	Neonate mothers	Screening due to pre-partum hospitalization for premature labour	Culture (API20E system)	Nasal/ Nares	198	1	0.51 [0.01; 2.78]
Doudoulakakis et al., 2022	Greece	Jan/2014-Dec/2018	Medical doctor (general), Nurse	Post-outbreak investigation.	Culture, EUCAST guidelines using cefoxitin	Nasal/ Nares	37	6	16.22 [6.19; 32.01]
Fujimura et al., 2004	Japan	Jan/2001-Jun/2001	HCWs	Response to ongoing detection of MRSA in symptomatic neonates.	Culture, PCR	Nasal/ Nares	14	7	50.00 [23.04; 76.96]
Fujimura et al., 2004	Japan	Jan/2001-Jun/2001	Incubator, Sheet, Chart, Nasogastric tube, Floor, Doorknob, Tap, Bath, Table/chair, Telephone, Switch, Cart, Scale, Stethoscope, White coat, Stationary	Response to ongoing detection of MRSA in febrile neonates.	Culture, PCR	Nasal/ Nares	85	45	52.94 [41.81; 63.87]
Gad et al., 2021	Egypt	Jan/2019-Dec/2019	Neonate mothers	Screening due to preterm neonate admission.	Culture	Expressed breast milk	118	2	1.69 [0.21; 5.99]
Keilman et al., 2021	United States of America	Unclear/Not reported	Baby Isolette Station, Communal Equipment, and Physical Plant	Routine surveillance study	Culture	Surfaces sampled	46	5	10.87 [3.62; 23.57]
Li et al., 2017 (baseline	China, Hong	Jan/2009-Dec/2013	environmental surfaces of patient zone, including cot rails, stethoscope,	Pre- and post-intervention study for	Culture, PCR	Surfaces sampled	100	44	44.00 [34.08; 54.28]

period (no intervention))	Kong SAR		infant incubator, monitor, syringe pumps, and drawer handles	cleaning measures					
Li et al., 2017 (intervention (environmental cleaning measures) period)	China, Hong Kong SAR	Jan/2009-Dec/2013	environmental surfaces of patient zone, including cot rails, stethoscope, infant incubator, monitor, syringe pumps, and drawer handles	Pre- and post-intervention study for cleaning measures	Culture, PCR	Surfaces sampled	800	20	2.50 [1.53; 3.83]
Nubel et al., 2013	Germany	Feb/2010-Aug/2010	HCWs	Routine surveillance study	Culture, PCR	Nasopharyngeal	160	2	1.25 [0.15; 4.44]
Okedo-Alex et al., 2020	Nigeria	Unclear/Not reported	HCWs	Routine surveillance study	Culture; Disk diffusion method using cefoxitin	Nasal/ Nares, Fingernail	51	9	17.65 [8.40; 30.87]
Richter et al., 2018	Germany	2016	HCWs	Staff testing followed increased rate of MSSA colonization	Culture	Buccal, Nasal/ Nares	128	2	1.56 [0.19; 5.53]
Sakai et al., 2020	Brazil	Jan2014-Feb2018	Neonate mothers	Routine surveillance study	Culture, Disk diffusion method CLSI guidelines	Axilla, Nasal/ Nares, Rectum, Inguinal, Oral	433	6	1.39 [0.51; 2.99]
Singh et al., 2018	India	Unclear/Not reported	Outer surface of the patients' files	Routine surveillance study	Culture, Disk diffusion method CLSI guidelines	Surfaces sampled	70	0	0.00 [0.00; 5.13]
Thomas et al., 2019 (Pre Intervention (Hand hygiene), Before Hand Wash)	India	Jul/2013-Aug/2013	Medical doctor (general), Nurse,	Pre- and post-hand hygiene intervention study	Culture	Hands	34	9	26.47 [12.88; 44.36]
Thomas et al., 2019 (Pre Intervention	India	Jul/2013-Aug/2013	Medical doctor (general), Nurse,	Pre- and post-hand hygiene	Culture	Hands	34	8	23.53 [10.75; 41.17]

(Hand hygiene), After Hand Wash)				intervention study					
Thomas et al., 2019 (Post Intervention (Hand hygiene), Before Hand Wash)	India	Jul/2013-Aug/2013	Medical doctor (general), Nurse,	Pre- and post-hand hygiene intervention study	Culture	Hands	34	0	0.00 [0.00; 10.28]
Thomas et al., 2019 (Post Intervention (Hand hygiene), After Hand Wash)	India	Jul/2013-Aug/2013	Medical doctor (general), Nurse,	Pre- and post-hand hygiene intervention study	Culture	Hands	34	0	0.00 [0.00; 10.28]

Table S4: Risk of bias assessment

Author, Year (Year of publication)	Was the study's target population a close representation of the national population in relation to MRSA	Was the sampling frame a true or close representation of the target population?	Was some form of random selection used to select the sample, OR was acensus undertaken?	Were data collected directly from the subjects (as opposed to a proxy)?	Was an acceptable inclusion criteria definition used in the study?	Did the author calculate and respect the expected sample size?	Was the MRSA detection assay shown to have reliability and validity?	Was the same mode of data collection used for all subjects?	Was the length of the study period > or = 1 year?	Were the numerator(s) and denominator(s) for the MRSA prevalence?	Risk of bias	Study participants

	prevalen ce?											
Adil A, 2010	No	Yes	No	Yes	Yes	No	Yes	Yes	No	Yes	Moderate risk of bias	HCWs
Bhatta, 2021	No	Yes	No	Not applicab le	Yes	No	Yes	Yes	Unclear	Yes	Moderate risk of bias	Environmental samples
Darwish, 2022	No	Yes	No	Yes	Yes	No	Yes	Yes	Unclear	Yes	Moderate risk of bias	HCWs; Neonate mothers
Davies, 2000	No	Yes	No	Not applicab le	Yes	No	Yes	Yes	No	Yes	Moderate risk of bias	Environmental samples
Denkel, 2014	No	Yes	No	Yes	Yes	No	Yes	Yes	Yes	Yes	Low risk of bias	Neonate mothers
Doudoulakak is, 2022	No	Yes	No	Yes	Yes	No	Yes	Yes	Yes	Yes	Low risk of bias	HCWs
Fujimura, 2004	No	Yes	No	Yes	Yes	No	Yes	Yes	No	Yes	Moderate risk of bias	HCWs; Environmental samples
Gad, 2021	No	Yes	No	Yes	Yes	No	Yes	Yes	Yes	Yes	Low risk of bias	Neonate mothers
Keilman, 2021	No	Yes	No	Not applicab le	Yes	No	Yes	Yes	No	Yes	Moderate risk of bias	Environmental samples
Li, 2017	No	Yes	No	Not applicab le	Yes	No	Yes	Yes	Yes	Yes	Moderate risk of bias	Environmental samples
Nubel, 2013	No	Yes	No	Yes	Yes	No	Yes	Yes	No	Yes	Moderate risk of bias	HCWs
Okedo-Alex, 2020	No	Yes	No	Yes	Yes	No	No	Yes	Unclear	Yes	Moderate risk of bias	HCWs
Richter, 2018	No	Yes	No	Yes	Yes	No	Yes	Yes	No	Yes	Moderate risk of bias	HCWs
Sakai, 2020	No	Yes	No	Yes	Yes	No	Yes	Yes	Yes	Yes	Low risk of bias	Neonate mothers
Singh, 2018	No	Yes	Yes	Not applicab le	Yes	No	Yes	Yes	No	Yes	Moderate risk of bias	Environmental samples

Thomas, 2019	No	Yes	No	Yes	Yes	No	Yes	Yes	No	Yes	Moderate risk of bias	HCWs

Figure S1: Funnel chart for publications of the proportion of MRSA carriage in neonate mothers in neonatal intensive care units

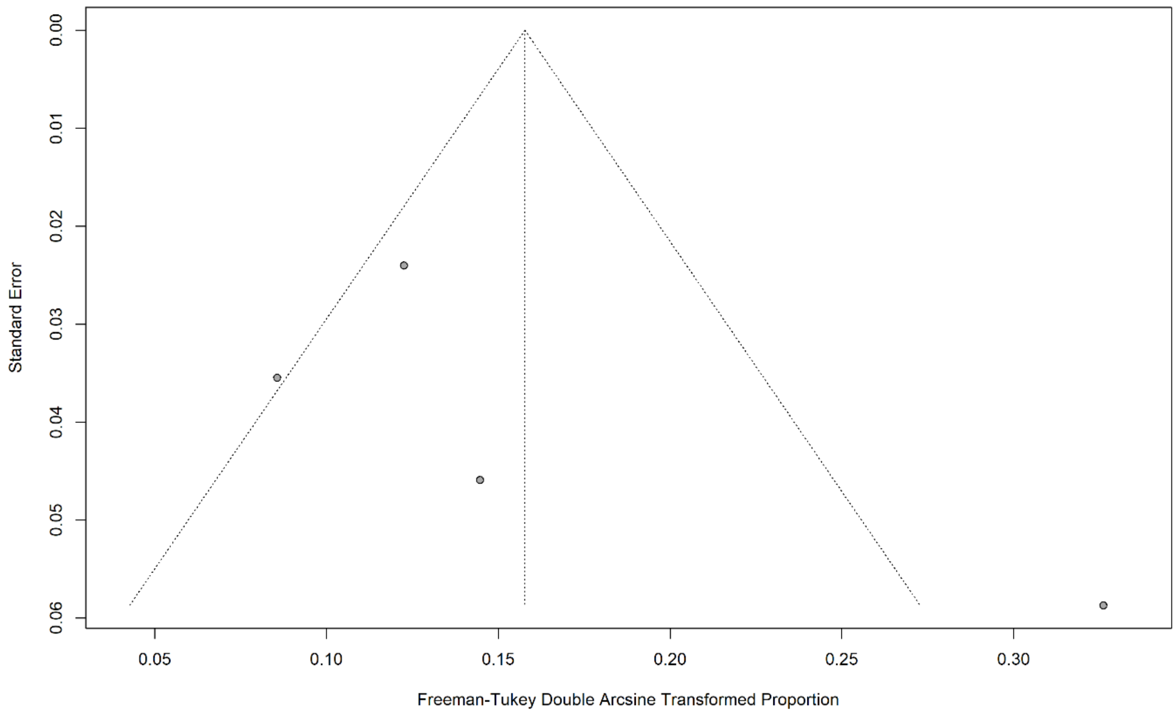

P Egger test: 0.315

Figure S2: Funnel chart for publications of the proportion of MRSA carriage in healthcare workers in neonatal intensive care units

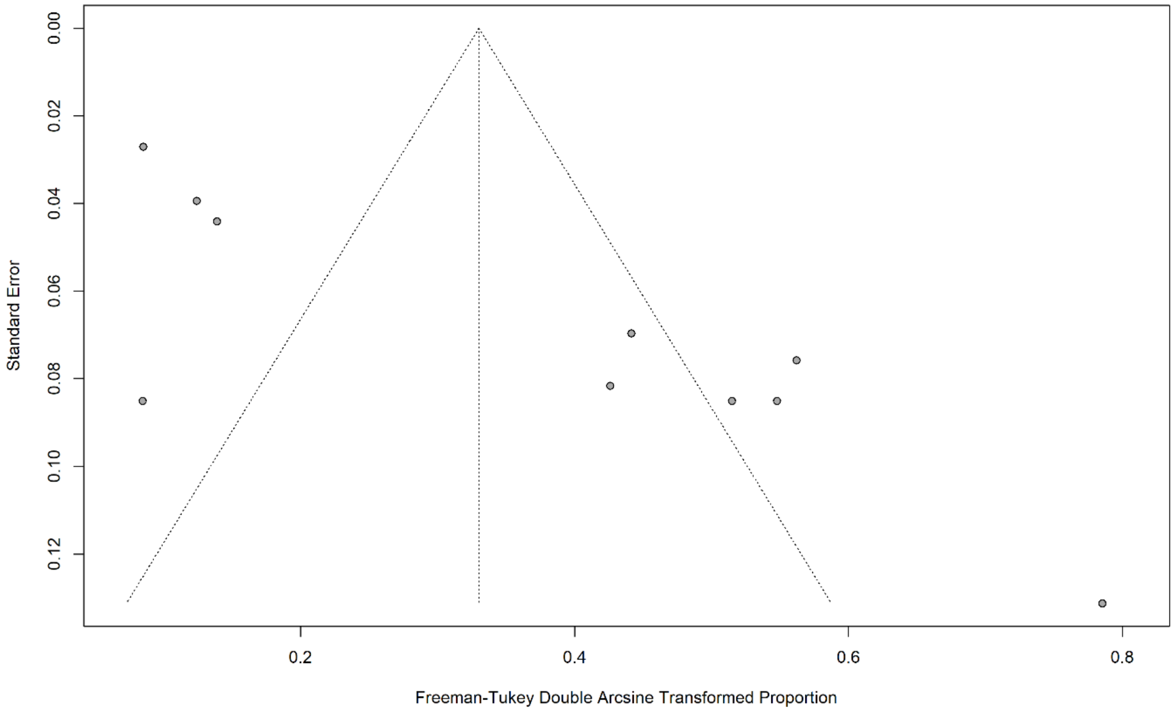

P Egger test: 0.004

Figure S3: Funnel chart for publications of the proportion of MRSA carriage in environmental surfaces in neonatal intensive care units

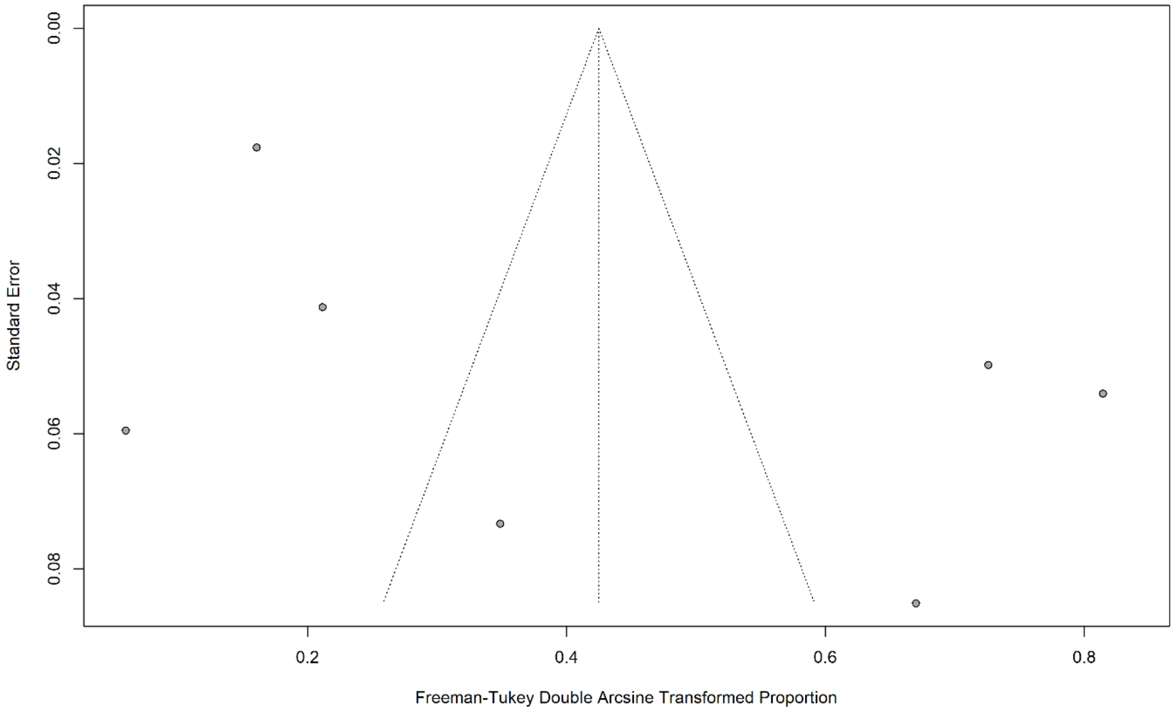

P Egger test: 0.134
